# Re-examining the pain–depression vicious cycle in knee osteoarthritis: a random-intercept cross-lagged panel analysis

**DOI:** 10.1186/s12891-026-10439-2

**Published:** 2026-09-05

**Authors:** Yannick Bruns, Hendrik Fahlbusch

**Affiliations:** 1 Department of Anesthesiology and Intensive Care Medicine, Pain Clinic, Hannover Medical School, Carl-Neuberg-Str. 1, Hannover, 30625 Germany; 2 Department of Orthopaedic and Trauma Surgery, Regio Kliniken, Fahltskamp 74, Pinneberg, 25421 Germany

**Keywords:** Osteoarthritis, Knee, Pain, Depression, Longitudinal, Random-intercept cross-lagged panel model, Within-person, Osteoarthritis Initiative

## Abstract

**Background:**

Pain and depression frequently co-occur in knee osteoarthritis (OA), traditionally viewed as a vicious cycle in which each worsens the other over time. However, conventional cross-lagged models cannot separate stable differences between individuals from real year-to-year changes within a person. We therefore used a random-intercept cross-lagged panel model (RI-CLPM) in the Osteoarthritis Initiative (OAI).

**Methods:**

Of 4,796 enrolled OAI participants with, or at risk of, knee OA, 4,638 contributed data across four annual waves from months 12 to 48. The RI-CLPM was applied to knee pain (WOMAC scale, worse knee) and depressive symptoms (CES-D scale), using full-information maximum likelihood. We contrasted it with a traditional model (CLPM), replicated it in a later six-wave window (months 36 to 96), and tested moderation by sex, arthritis severity, baseline pain and baseline depression, alongside covariate-adjusted and sensitivity analyses. Sensitivity analyses used a cognitive–affective CES-D subscale excluding somatic items, alternative model constraints and an alternative standardisation.

**Results:**

The RI-CLPM fitted well and the between-person trait correlation was substantial (standardised *r* = 0.39, *p* < 0.001), whereas within-person cross-lagged paths were small and non-significant in both directions (pain to depression standardised beta = 0.019, 95% CI -0.010 to 0.048; depression to pain beta = 0.009, 95% CI -0.019 to 0.036). A classical CLPM on identical data showed significant reciprocal paths (both beta approximately 0.08) but fitted poorly; separating the random intercepts reduced the cross-lagged coefficients by roughly 77–89%. The pattern was reproduced in the second window, unchanged by covariates, and no moderator test survived correction for multiple testing. Using a cognitive–affective CES-D subscale reduced both cross-lagged paths further (0.014 and − 0.001) and the trait correlation to 0.34.

**Conclusions:**

In knee OA, the link between pain and depression was mainly explained by stable differences between individuals. At annual intervals we found limited evidence for reciprocal within-person effects, which is not evidence that none exist; shorter-term dynamics were not estimable. Depressive symptoms should still be assessed, but the findings do not support an automatic year-to-year feedback loop, and do not show whether treating one symptom improves the other.

**Supplementary Information:**

The online version contains supplementary material available at https://doi.org/10.1186/s12891-026-10439-2.

## Background

Knee osteoarthritis (OA) is a major and growing contributor to pain and disability worldwide [1], and depressive symptoms are common among people with knee OA and are associated with poorer outcomes [2]. A reciprocal association between pain and depression has been reported, with longitudinal associations documented in both directions across chronic-pain and OA populations [3–6]. At the disease level, bidirectional associations between incident knee OA and depressive symptoms have also been reported in a population-based cohort [7]. These findings have contributed to the hypothesis that pain and depressive symptoms reinforce one another over time, a vicious cycle in which each amplifies the other. This framing is common in OA research and has also been supported by analyses of the Osteoarthritis Initiative (OAI) [2, 8], and underlies the rationale for integrated psychological and physical management of knee OA. As used here, a vicious cycle denotes a self-reinforcing feedback loop within an individual, in which a change in one symptom produces a change in the other that in turn feeds back on the first. Reciprocal cross-lagged associations are a necessary but not a sufficient condition for such a loop, since they establish temporal prediction rather than a reinforcing mechanism.

However, these reviews also note that the bidirectional relationship remains inconclusive [2]. It remains uncertain whether this association reflects a dynamic, mutually reinforcing process within individuals over time, as the vicious-cycle metaphor implies, or whether it largely reflects stable differences between individuals. Distinguishing these is a methodological question that the existing evidence base has not resolved.

Much of the available evidence for a genuinely dynamic, mutually reinforcing process is based on cross-sectional associations, classical cross-lagged panel models (CLPMs), or simple lagged regressions [3–6]. These approaches share a well-recognised limitation [9, 10]: they do not separate stable, time-invariant differences between people (between-person, or trait, variance) from fluctuations within a person over time (within-person, or state, variance). If these two sources of variation are not separated, stable differences between people may be mistaken for a dynamic process within individuals, in which a change in one drives a later change in the other. Examining this question therefore requires a method that isolates within-person change.

The random-intercept cross-lagged panel model (RI-CLPM) was developed for precisely this purpose [9]. By adding a random intercept for each construct, it partitions stable between-person differences from within-person dynamics and estimates the cross-lagged (reciprocal) paths at the within-person level. Applying the RI-CLPM has repeatedly shown that reciprocal effects which appear robust in a classical CLPM can attenuate substantially, or disappear, once stable between-person differences are accounted for [9, 10]. RI-CLPMs of pain and depressive symptoms have been reported in veteran and population-based ageing cohorts [11–14], but to our knowledge no such analysis has been conducted in the OAI or in any knee-specific OA cohort. Given the frequent use of reciprocal pain–depression models in knee OA and their connection to OAI-based evidence, a within-person analysis in this cohort is warranted. The closest prior work within the OAI used marginal structural models to estimate the effect of depressive-symptom histories on subsequent knee pain under assumptions about time-varying confounding [8]. That approach asks what would follow from a given exposure history. The present analysis asks a different question, namely at which level of variation the observed association resides. The two are complementary rather than competing. The distinction also carries a practical consequence. A predominantly within-person association would imply that change in one domain gives early warning about the other; a predominantly between-person association shifts the clinical expectation towards managing a persistent comorbid burden.

### Aim and hypotheses

We examined whether the pain–depression association in knee OA reflects a dynamic within-person process or predominantly stable between-person differences, using an RI-CLPM in the OAI. We tested four hypotheses. H1: higher pain than a person’s own usual level predicts subsequently elevated depressive symptoms (within-person pain → depression). H2: elevated depressive symptoms predict subsequently elevated pain (within-person depression → pain). H3: the stable between-person components of pain and depression are positively correlated (between-person association). H4 (exploratory): the within-person cross-lagged effects differ by sex, radiographic severity, baseline pain severity, or baseline depressive status. We additionally contrasted the RI-CLPM with a classical CLPM on identical data to determine whether the association was primarily attributable to between-person or within-person variation, and assessed robustness across a later, partially overlapping time window.

## Methods

### Reporting and design

This is a secondary analysis of prospectively collected longitudinal data from the Osteoarthritis Initiative (OAI) [15]. Reporting follows the STROBE recommendations for observational cohort studies [16]. The OAI was approved by the institutional review boards of the participating clinical sites and by the OAI Coordinating Center at the University of California, San Francisco, and all participants provided written informed consent. The present analysis used only de-identified public-use data and involved no new participant contact; further ethics details are given in the Declarations.

### Data source and participants

The OAI is a multicentre, prospective cohort study of knee osteoarthritis that enrolled 4,796 participants aged 45–79 years at four US clinical sites between February 2004 and May 2006, with annual follow-up. Participants were recruited into a progression subcohort (symptomatic radiographic knee OA at baseline), an incidence subcohort (at elevated risk of developing knee OA), and a small non-exposed control subcohort. The analytic sample therefore comprises adults with, or at elevated risk of, knee OA rather than an exclusively radiographic-OA sample; at the first analysed wave a substantial minority had no radiographic OA (KL < 2; see Table 1). Data are publicly available through the OAI repository. The present analysis used the clinical assessment datasets, which contain the self-reported pain and depression measures, supplemented by central radiographic readings for one moderator (see below). Data were obtained from the OAI repository at the NIMH Data Archive on 7 March 2026. The specific datasets used were AllClinical00, AllClinical01, AllClinical03 and AllClinical05 to AllClinical10 (versions 0.2.3, 1.2.2, 3.2.1, 5.2.1, 6.2.1, 7.2.1, 8.2.2, 9.2.1 and 10.2.2), together with Enrollees (version 25) and MeasInventory (version 11).

**Table 1:** Baseline and first analysed wave characteristics of the enrolled Osteoarthritis Initiative cohort (*N* = 4,796; primary analytic sample *n* = 4,638)

Characteristic	Value
Age, years, mean (SD)	61.2 (9.2)
Female, *n* (%)	2804 (58.5)
Body mass index, kg/m2, mean (SD)	28.6 (4.8)
Subcohort: progression / incidence / control, %	29.0 / 68.5 / 2.5
KL grade 0 / 1 / 2 / 3 / 4, % (worse knee)	26.2 / 14.6 / 28.4 / 18.6 / 6.1
KL grade missing, %	6.0
WOMAC pain, worse knee, mean (SD)	3.0 (3.6)
WOMAC pain, worse knee, median (IQR); skewness	2 (0–5); 1.42
CES-D, mean (SD)	6.6 (7.0)
CES-D, median (IQR); skewness	4 (1–9); 1.93
Depressed at baseline (CES-D ≥ 16), *n* (%)	480 (10.0)

*KL* Kellgren-Lawrence, *WOMAC* Western Ontario and McMaster Universities Osteoarthritis Index (worse knee, range 0–20), *CES-D* Center for Epidemiologic Studies Depression Scale (range 0–60). Age, sex, BMI, subcohort, KL grade and depressed-at-baseline status are from the OAI baseline visit (V00); WOMAC pain and CES-D are shown at the first analysed wave (V01, 12 months)

### Analytic sample

All enrolled participants with at least one available pain or depression measurement during the relevant observation window were eligible. Missing data were handled with full-information maximum likelihood (FIML; see Statistical analysis), so cases with partial data contributed to estimation. Of the 4,796 enrolled participants, 4,638 contributed at least one pain or depression observation within the primary window and were included in the primary FIML model; 158 had no usable pain or depressive-symptom observation in this window and therefore contributed no information. Complete data across all four primary waves (all eight panel variables) were available for 3,525 participants. Among the 4,638 participants analysed, 3,925 contributed data at all four waves, 337 at three, 212 at two and 164 at a single wave. Missing observations arise from missed visits, incomplete questionnaire return and attrition, which the OAI public-use data do not allow to be distinguished. Sample formation and wave-specific availability are shown in Additional file 1, Table S1.

### Measures

#### Knee pain

Knee pain was measured with the Western Ontario and McMaster Universities Osteoarthritis Index (WOMAC) pain subscale (range 0–20, higher = worse) [17]. Because the OAI records pain for each knee separately, the analysis used the score of the more symptomatic (worse) knee at each visit, defined as the higher of the two knee-specific WOMAC pain scores. This score represents overall knee-pain burden; because the more symptomatic knee may differ between visits, it indexes overall burden rather than a single fixed joint, a point considered in the Discussion.

#### Depressive symptoms

Depressive symptoms were measured with the 20-item Center for Epidemiologic Studies Depression Scale (CES-D; summed range 0–60, higher = more symptoms) [18]. The CES-D is a validated screening measure of depressive symptom burden, not a clinical diagnosis. The conventional screening threshold (CES-D ≥ 16) was used only to define a moderator subgroup, not to dichotomise the outcome. Because somatic CES-D items may overlap with the physical consequences of knee pain, a sensitivity analysis used a cognitive–affective subscale in place of the total score. Item-level responses were used to reconstruct the subscales of the original instrument [18]. The seven somatic and retarded-activity items (items 1, 2, 5, 7, 11, 13 and 20) were removed, leaving thirteen cognitive–affective items. Subscale scores were calculated when at least 80 per cent of the respective items were answered.

#### Timing of measurements

Both WOMAC pain and CES-D were collected at the OAI’s annual visits. The primary model used four approximately equidistant annual waves spanning 12 to 48 months (visits V01, V03, V05, V06; i.e., 12, 24, 36 and 48 months). A second, later window spanning 36 to 96 months (six consecutive annual waves, partially overlapping the primary window at 36 and 48 months) was used for robustness (see Statistical analysis). Sub-annual intervals were not analysable because the intermediate OAI visits do not include the CES-D; this constraint is considered in the Discussion. All modelled intervals are therefore twelve months. The CES-D is available at nine OAI visits, namely baseline and 12, 24, 36, 48, 60, 72, 84 and 96 months.

#### Moderators

Four planned moderators were examined: sex (male/female); radiographic OA severity, defined as the Kellgren–Lawrence (KL) grade of the worse knee at the OAI baseline visit (V00) from central readings, dichotomised as radiographic OA present (KL ≥ 2) versus absent (KL < 2); baseline pain severity, defined as the worse-knee WOMAC pain score at the OAI baseline visit (V00) and dichotomised at the sample median of 2 points; and baseline depressive status (CES-D ≥ 16 versus < 16 at the OAI baseline visit, V00). Baseline pain was measured before the first RI-CLPM wave to avoid defining moderator groups using a panel variable that is itself part of the model. The exact median of baseline WOMAC pain in the analytic sample was 2 points (interquartile range 0 to 5; mean 3.42, SD 3.68; range 0 to 20). The lower group comprised 2,427 participants (median 0, interquartile range 0 to 1) and the higher group 2,211 (median 6, interquartile range 4 to 8). Because a median split is an arbitrary reduction of a continuous variable, the baseline-pain moderation was repeated using three further groupings: any pain versus none, the upper third versus the remainder, and a three-group split at 0, 1 to 3 and ≥ 4 points.

### Statistical analysis

#### Primary model: random-intercept cross-lagged panel model

The longitudinal relationship between pain and depression was estimated with a random-intercept cross-lagged panel model (RI-CLPM) as specified by Hamaker and colleagues [9]. The RI-CLPM decomposes each repeated measure into (i) a stable, time-invariant random intercept capturing between-person (trait) differences, and (ii) a time-varying within-person component. Two random intercepts (one for pain, one for depression) capture stable individual differences and their between-person association; following the standard parameterisation, the random intercepts were specified orthogonal to the within-person components of the first wave. The within-person components are linked over time by autoregressive paths (each construct predicting its own subsequent within-person deviation) and cross-lagged paths (each construct predicting the other’s subsequent within-person deviation). The cross-lagged paths were the parameters of primary interest. They indicate whether pain above a person’s usual level predicts depressive symptoms above that person’s usual level at the next wave, and vice versa. Within each wave, the residual within-person components of pain and depressive symptoms were allowed to correlate. This captured their contemporaneous (same-wave) within-person association alongside the lagged cross-paths.

Observed variables were z-standardised separately within each wave, using the mean and standard deviation of the cases available at that wave, so that path coefficients are interpretable on a common standardised scale. Because this removes differences in variance between waves rather than modelling them, the primary model was also re-estimated after standardising across all waves jointly, which is equivalent to using the raw scale. Autoregressive and cross-lagged paths were constrained to equality across waves to yield parsimonious, stable estimates of the average lagged effect; this constraint was relaxed in sensitivity analysis. Models were estimated by maximum likelihood with FIML for missing data, using all available cases. Model fit was evaluated with the model chi-square, the comparative fit index (CFI), the Tucker–Lewis index (TLI), the root mean square error of approximation (RMSEA) and the standardised root mean square residual (SRMR). These indices were selected because they are sensitive to different aspects of misspecification, and because the model chi-square alone is of limited value at this sample size. Commonly used reference values (approximately CFI ≥ 0.95, RMSEA ≤ 0.06, SRMR ≤ 0.08) were treated as conventional benchmarks rather than decision thresholds and were interpreted jointly, alongside the overall pattern of fit [19]. The primary model estimated 27 free parameters, of which 8 were intercepts, leaving 17 degrees of freedom; all estimated parameters are reported in Additional file 1, Table S2. In a covariate-adjusted model, age, sex and body mass index, all measured at the OAI baseline visit, were added as predictors of both random intercepts to account for stable confounders of the between-person association. They entered at the between-person level only, being time-invariant or nearly so across the analytical window and therefore carrying no within-person variance.

#### Contrast with the classical cross-lagged panel model

**Fig. 1:**
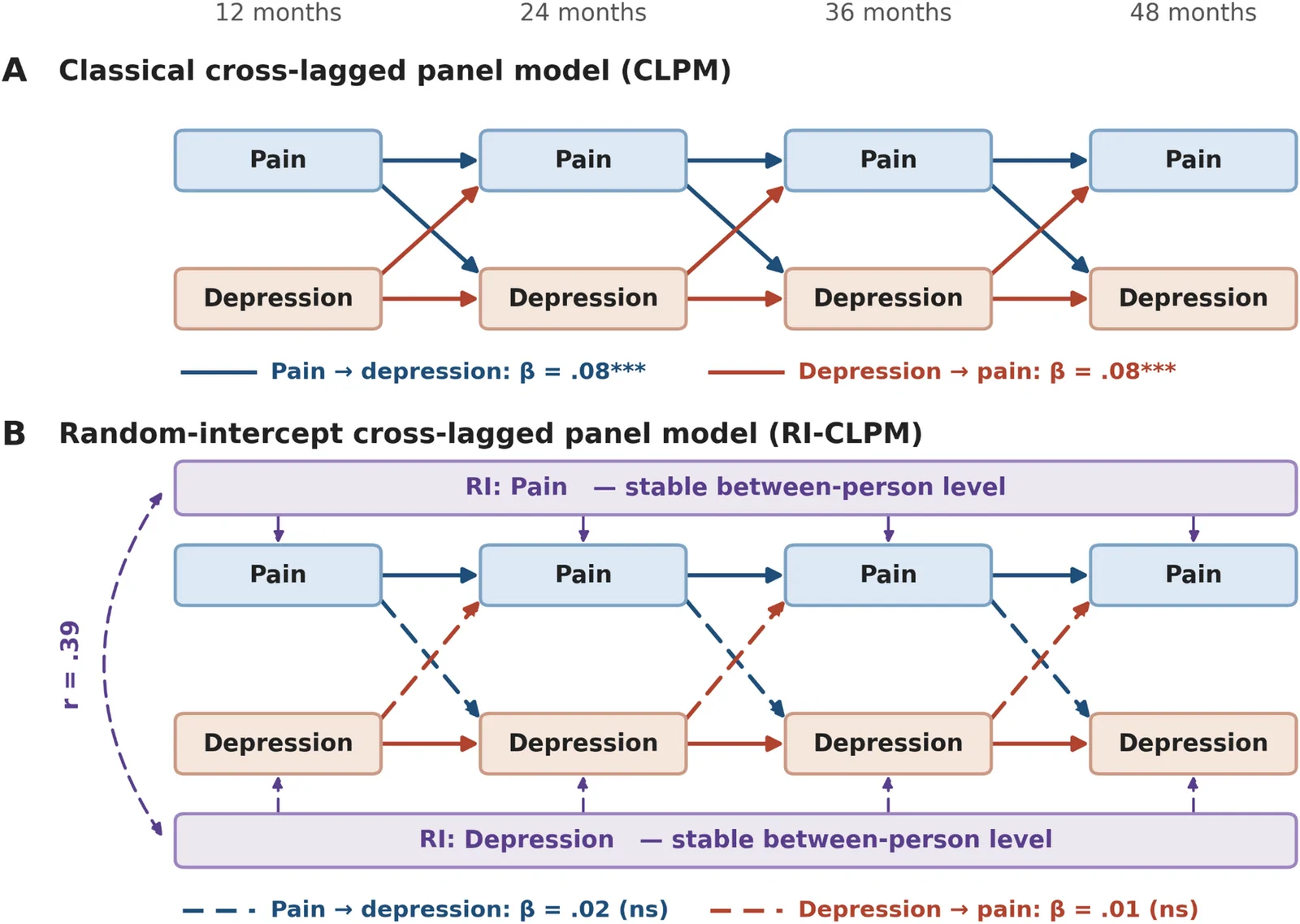
Conceptual comparison of the classical and random-intercept cross-lagged panel models. Both panels show the same two constructs measured at the four annual assessments analysed here, linked by autoregressive (horizontal) and cross-lagged (diagonal) paths. In the RI-CLPM (panel **B**), two random intercepts (RI: Pain, RI: Depression) additionally capture stable between-person differences and are correlated with one another. The RI-CLPM requires at least three measurement occasions to separate between-person from within-person variation, and is therefore shown with the full set of analysed waves. Autoregressive and cross-lagged paths were constrained to equality across waves, so a single estimate is shown for each path. Standardised estimates; primary window, months 12–48; *n* = 4,638. CLPM, Cross-lagged panel model; RI, Random intercept; RI-CLPM, Random-intercept cross-lagged panel model

For comparison, a classical cross-lagged panel model (CLPM) with the same autoregressive and cross-lagged structure but without the two random intercepts was estimated on the same data. The cross-lagged coefficients from the two models were compared directly to quantify the extent to which the reciprocal estimates in the classical model are attributable to stable between-person differences. The comparison is descriptive: it distinguishes between-person from within-person variation and is not intended as a claim that earlier CLPM-based findings are invalid. Figure 1 illustrates conceptually how the two models partition the pain-depression association.

#### Robustness across a second time window

To assess dependence on the chosen waves, the primary RI-CLPM was re-estimated on a later sequence of six consecutive annual waves spanning 36 to 96 months, partially overlapping the primary window (36 and 48 months) in the same participants. Consistency of the cross-lagged coefficients, the between-person trait correlation and model fit across the two windows was taken as evidence of robustness to the choice of waves. The primary window was chosen because the baseline visit was reserved for defining the moderator variables, and because four consecutive equidistant waves identify the RI-CLPM while keeping the number of estimated parameters modest. Together the two windows cover all annual assessments from 12 to 96 months.

### Moderation analysis

For each of the four moderators, a multigroup RI-CLPM was estimated and a likelihood-ratio test (2 degrees of freedom) compared a model with cross-lagged paths freely estimated across groups against a model constraining them to equality. A significant test would indicate that the within-person cross-lagged effects differ across the moderator subgroups. Group-specific cross-lagged estimates are reported alongside each test.

### Sensitivity analyses

Planned sensitivity analyses comprised: (i) complete-case estimation compared with FIML; (ii) freely estimated (rather than equality-constrained) lagged paths; and (iii) the classical CLPM as a contrasting specification. Further analyses comprised: (iv) a series of nested models relaxing the equality constraints separately and jointly, compared by likelihood-ratio test and by the fit indices above together with AIC and BIC; (v) the cognitive–affective CES-D subscale in place of the total score; (vi) pooled rather than wave-specific standardisation; and (vii) the alternative groupings of baseline pain. Analyses were conducted in R version 4.6.0 using the lavaan package (version 0.6-21) [20].

## Results

### Sample characteristics

The analytic cohort comprised 4,796 enrolled participants, of whom 4,638 contributed pain or depression data within the primary window under FIML; 3,525 had complete data across all four primary waves. Baseline characteristics are shown in Table 1.

### Primary RI-CLPM and contrast with the classical CLPM

**Fig. 2:**
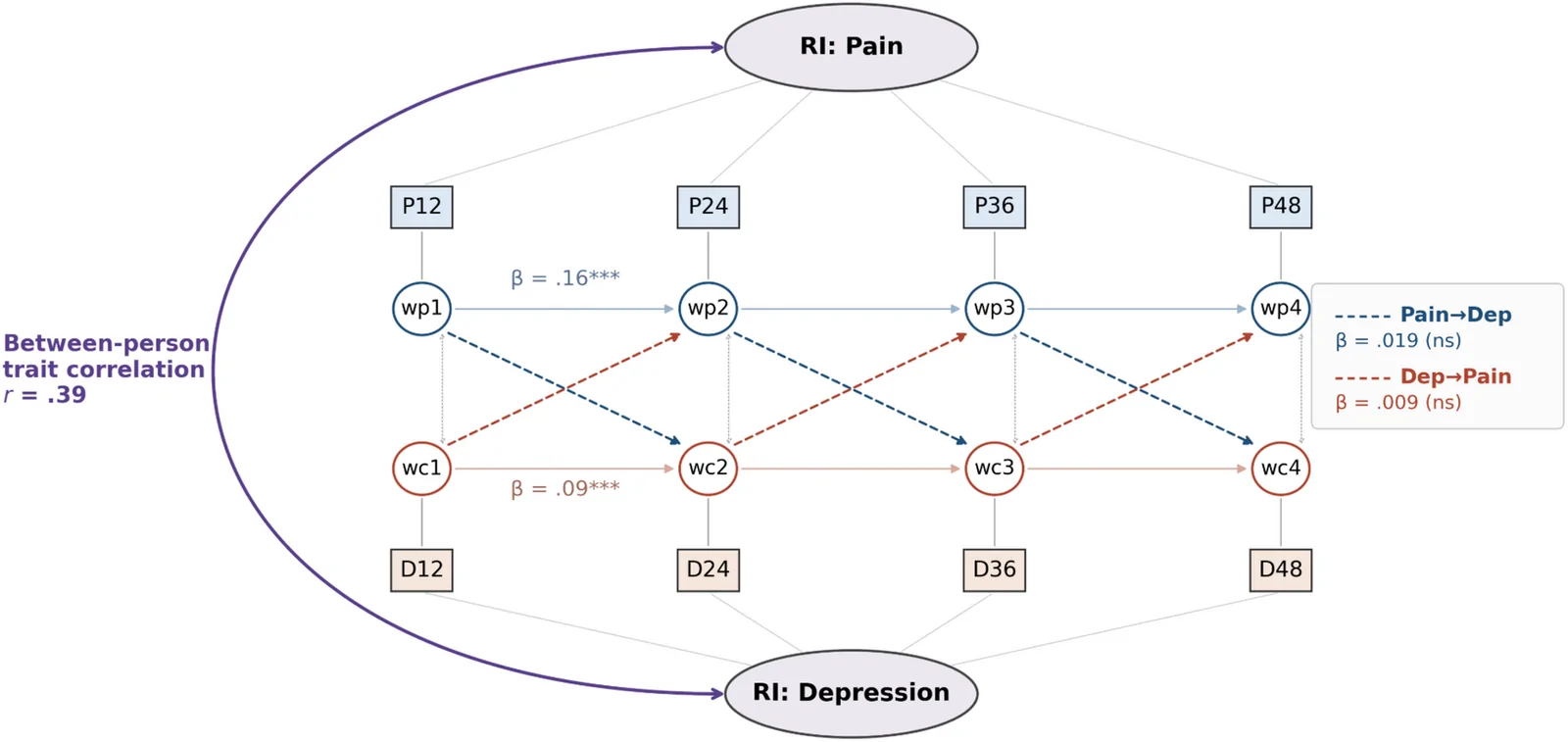
Random-intercept cross-lagged panel model for WOMAC pain and CES-D depressive symptoms. Each observed score (P = WOMAC pain, D = CES-D) is decomposed into a stable between-person Random Intercept (RI) and a Within-person Component (wp, wc). Solid arrows, autoregressive paths; dashed coloured arrows, within-person cross-lagged paths; dotted arrows, within-wave correlations; curved connector on the left, between-person trait correlation. Standardised estimates; primary window, months 12–48; *n* = 4,638. CES-D, Center for Epidemiologic Studies Depression Scale; RI, Random intercept; WOMAC, Western Ontario and McMaster Universities Osteoarthritis Index

The RI-CLPM fitted the data well (chi-square = 18.4, df = 17, *p* = 0.36; CFI = 1.00; RMSEA = 0.004; SRMR = 0.007). The between-person random intercepts of pain and depression were positively correlated (standardised *r* = 0.39, *p* < 0.001), while the within-person cross-lagged paths were small and non-significant in both directions (pain to later depression: standardised beta = 0.019, 95% CI -0.010 to 0.048, *p* = 0.21; depression to later pain: standardised beta = 0.009, 95% CI -0.019 to 0.036, *p* = 0.54). In the classical CLPM on identical data, both cross-lagged paths were substantially larger and significant (standardised beta = 0.079 and 0.078, both *p* < 0.001), and that model fitted poorly (CFI = 0.88, RMSEA = 0.15, SRMR = 0.08); after separating stable between-person differences, the cross-lagged coefficients were reduced by approximately 77% and 89%, respectively. Within-person residuals showed a small contemporaneous correlation (standardised *r* = 0.044 to 0.071 across waves; nominal *p* < 0.05 in three of the four waves, without correction for multiple testing). Estimates are shown in Table 2, and the model structure with standardised estimates is shown in Fig. 2. The corresponding unstandardised coefficients were 0.018 (SE 0.014) and 0.009 (SE 0.014), in within-wave standard deviation units. On the original scales this corresponds to 0.037 CES-D points per WOMAC point and 0.004 WOMAC points per CES-D point.

**Table 2:** Standardised cross-lagged estimates: classical CLPM versus RI-CLPM (primary window, 12–48 months)

Path / parameter	Classical CLPM	RI-CLPM
Pain -> later depression	+ 0.079***; 95% CI 0.065, 0.093; B = 0.079 (SE 0.007)	+ 0.019 (ns); 95% CI -0.010, 0.048; B = 0.018 (SE 0.014)
Depression -> later pain	+ 0.078***; 95% CI 0.064, 0.092; B = 0.078 (SE 0.007)	+ 0.009 (ns); 95% CI -0.019, 0.036; B = 0.009 (SE 0.014)
Attenuation (RI-CLPM vs. CLPM)	-	-77% (pain -> depression); -89% (depression -> pain)
Between-person trait correlation	-	+ 0.39***
Contemporaneous within-person r	-	+ 0.044 to + 0.071
Model fit (CFI / RMSEA / SRMR)	0.88 / 0.15 / 0.08	1.00 / 0.004 / 0.007

Standardised coefficients (std.all); 95% confidence intervals for the cross-lagged paths are from the standardised solution. Unstandardised cross-lagged coefficients (B, with standard errors) were constrained to equality across waves and are expressed in within-wave standard deviation units; fully standardised estimates varied slightly across transitions (pain to depression standardised beta 0.018–0.019). The complete set of unstandardised parameters is given in Additional file 1, Table S2. ns, not significant; ***p < 0.001. Attenuation = reduction of the cross-lagged coefficient from CLPM to RI-CLPM. Contemporaneous within-person r ranged from 0.044 to 0.071 across waves (nominal *p* < 0.05 in three of four waves, uncorrected). The random intercepts were specified orthogonal to the first-wave within-person components, following the standard RI-CLPM parameterisation. Estimated in lavaan 0.6-21 with full-information maximum likelihood (*n* = 4,638)

Concurrent correlations between WOMAC pain and the CES-D were of similar magnitude at every wave (Pearson *r* = 0.267, 0.262, 0.262 and 0.283 at 12, 24, 36 and 48 months; all *p* < 0.001), with similar Spearman coefficients (0.234 to 0.251). Wave-specific and cross-wave correlations are given in Additional file 1, Table S3.

### Depressive symptoms excluding somatic items

The somatic CES-D subscale correlated more strongly with WOMAC pain than the cognitive–affective subscale at every wave (*r* = 0.285 to 0.311 versus *r* = 0.201 to 0.236). When the RI-CLPM was re-estimated with the cognitive–affective subscale in place of the total score, model fit was comparable (chi-square = 19.3, df = 17, *p* = 0.31; CFI = 1.00; RMSEA = 0.005; SRMR = 0.007) and both cross-lagged paths became smaller rather than larger (standardised beta = 0.014, *p* = 0.35, and − 0.001, *p* = 0.95). The between-person trait correlation fell from 0.39 to 0.34. Full estimates are given in Additional file 1, Table S4.

### Robustness across a second time window

Re-estimation over six consecutive annual waves (36–96 months) reproduced the pattern: small, non-significant within-person cross-lagged paths and a stable between-person trait correlation (Table 3).

**Table 3:** Within-person cross-lagged estimates across two time windows

Model	Pain-> Depr	Depr-> Pain	Trait *r*
Primary (12–48 m, 4 waves)	+ 0.019 ns	+ 0.009 ns	+ 0.39
Robustness (36–96 m, 6 waves)	+ 0.012 ns	+ 0.013 ns	+ 0.41

Standardised coefficients. *ns* Not significant. RI-CLPM with FIML in lavaan 0.6-21 (primary *n* = 4,638; robustness *n* = 4,467)

### Moderation analysis

There was no evidence of moderation by sex (likelihood-ratio chi-square = 1.84, df = 2, *p* = 0.399), radiographic OA status (chi-square = 3.37, df = 2, *p* = 0.186), or baseline pain severity (chi-square = 0.75, df = 2, *p* = 0.687). The baseline-depression comparison was nominally significant (chi-square = 6.29, df = 2, *p* = 0.043), with larger depression-to-pain estimates among participants with CES-D scores ≥ 16 (Table 4). After correction for the four exploratory moderator analyses (Bonferroni-adjusted threshold 0.0125), this comparison was no longer significant. It was also based on a smaller subgroup (*n* = 480) with wide confidence intervals; we therefore regard it as hypothesis-generating rather than as evidence of robust effect modification. The baseline-pain comparison remained non-significant under all three alternative groupings (chi-square = 1.39, 0.24 and 2.60; *p* = 0.499, 0.886 and 0.627), with group-specific standardised estimates between − 0.014 and 0.038 (Additional file 1, Table S5).

**Table 4:** Multigroup moderation of within-person cross-lagged paths (likelihood-ratio tests, 2 df)

Moderator	LRT chi-square	*p*
Sex	1.84	0.399
Radiographic OA (KL ≥ 2)	3.37	0.186
Baseline pain at V00 (median split)	0.75	0.687
Baseline depression (CES-D ≥ 16)	6.29	0.043

Likelihood-ratio test, 2 df, comparing freely estimated versus equality-constrained cross-lagged paths across groups. Tests were exploratory and uncorrected. None remained significant under a Bonferroni-adjusted threshold of 0.0125. RI-CLPM multigroup models in lavaan 0.6-21 with FIML

### Covariate-adjusted and sensitivity analyses

With age, sex and body mass index added as covariates of the random intercepts, the within-person cross-lagged paths remained small and non-significant (pain to depression standardised beta = 0.020, *p* = 0.17; depression to pain beta = 0.009, *p* = 0.52); body mass index was the strongest correlate of the stable pain and depression intercepts. Results were stable across sensitivity analyses: complete-case estimation (*n* = 3,525) reproduced the null cross-lagged paths and the trait correlation (standardised *r* = 0.37), and a model with freely estimated rather than equality-constrained lags did not fit significantly better (likelihood-ratio test *p* = 0.41), with all individual cross-lagged paths remaining non-significant. A series of nested models, each relaxing one equality constraint relative to the primary specification, supported the constrained model. Relaxing the autoregressive paths did not improve fit (difference in chi-square = 5.36, df = 4, *p* = 0.253), nor did relaxing the cross-lagged paths (2.46, df = 4, *p* = 0.652) or both together (8.29, df = 8, *p* = 0.406). On the pooled scale the autoregressive constraint was rejected (16.13, df = 4, *p* = 0.003), those paths increasing across waves, whereas the cross-lagged constraint held on both scales (Additional file 1, Table S6). Pooled rather than wave-specific standardisation left the estimates essentially unchanged (pain to depression standardised beta = 0.018, *p* = 0.20; depression to pain 0.007, *p* = 0.63; trait correlation 0.39), with fit remaining good (Additional file 1, Table S7). Across all specifications examined, standardised cross-lagged coefficients ranged from − 0.030 to 0.045 and none reached significance.

## Discussion

### Principal findings

In this large OAI cohort, the association between pain and depressive symptoms was primarily located at the between-person level. The two random intercepts were substantially correlated, indicating that participants with higher average pain also tended to report higher average depressive symptoms. By contrast, the within-person cross-lagged effects were small and non-significant in both directions, providing limited evidence for reciprocal within-person effects at annual intervals. Because the OAI administers the CES-D only once a year, this finding speaks to year-to-year associations rather than to shorter-term processes; this pattern was reproduced in a later six-wave window spanning 36 to 96 months that partially overlapped the primary window. The cross-lagged coefficients were substantially smaller in the RI-CLPM than in the classical CLPM, which challenges the common interpretation of longitudinal pain-depression associations in knee OA as a self-reinforcing year-to-year process within individuals. Within the same wave, within-person deviations in pain and depressive symptoms were weakly correlated (nominal *p* < 0.05 in three of the four waves). The cross-lagged estimates, by contrast, were close to zero, with confidence intervals that were inconsistent with moderate or large year-to-year effects, while the between-person correlation was substantially larger. The same pattern held when the somatic CES-D items were removed, which indicates that the small residual association in the total score reflects shared somatic content in part rather than affective symptomatology.

### Interpretation

Clinically, this pattern suggests that pain and depressive symptoms tend to co-occur persistently rather than reinforce one another from year to year. This distinction matters, because the vicious-cycle framing is often used to support the expectation that reducing one symptom will, through a reciprocal dynamic, reduce the other; our data provided little evidence for such year-to-year carry-over. Shared stable factors may contribute to this co-occurrence, alongside small contemporaneous fluctuations in pain and mood, rather than a process in which each drives the other from one year to the next. This reading is consistent with within-person analyses of pain and depression in ageing cohorts [12]. The significant same-wave covariances indicate that the within-person components did capture contemporaneous covariation. This does not by itself establish sensitivity to all possible lagged effects, but the cross-lagged estimates were nonetheless precise, with confidence intervals that were inconsistent with moderate or large year-to-year effects. The estimates therefore provide little support for a clinically meaningful year-to-year cross-lagged effect, even though a null hypothesis test cannot establish the exact absence of an effect. The between-person correlation is likewise a description rather than an explanation. It is consistent with shared constitutional, socioeconomic or behavioural factors, with common inflammatory or central-sensitisation pathways, with response styles common to both instruments, and with reverse-causal histories predating the observation window. The RI-CLPM cannot distinguish among these.

### Comparison with previous work

Within the OAI, the nearest prior work used marginal structural models to estimate the dynamic effect of depressive symptoms on WOMAC knee pain and found higher pain with persistent depressive symptoms [8]; that analysis addressed the effect of depressive symptom histories on subsequent pain, not the between- versus within-person decomposition examined here, so the two are complementary rather than overlapping. Recent RI-CLPM analyses of pain and depression in ageing populations have yielded somewhat heterogeneous findings. In the Chinese CHARLS cohort a within-person effect of depression on later pain persisted while the reverse did not [11], and two large European and US ageing cohorts reported small but bidirectional within-person effects [12]. A home-dwelling older-adult cohort found a small pain-to-depression path [13], and a large US veteran cohort, using the same CLPM-versus-RI-CLPM contrast, likewise found that reciprocal effects diminished once stable between-person differences were separated [14]. Our knee-specific analysis differs in two respects. First, it uses a disease-specific pain measure (WOMAC) in a knee OA and at-risk sample rather than a general report of bodily pain in an unselected ageing population. Second, our estimates were close to zero in both directions, whereas small within-person associations remained in some of these cohorts. That reciprocal effects prominent in classical models attenuate under the RI-CLPM is itself an established pattern, demonstrated in methodological work on model choice [9, 10]. The present study extends this literature to a knee-specific, disease-focused setting by examining WOMAC pain in the OAI. Where a small within-person pain-to-depression effect has reached significance in another cohort (standardised beta around 0.04) [13], that estimate was small and fell within the confidence interval of our standardised estimate. Differences in interval length, population and pain measurement nonetheless limit direct comparison. Two differences are worth stating. The WOMAC pain subscale is knee-specific and activity-referenced, whereas the cohorts reporting small but non-zero within-person effects used general bodily-pain items. The OAI is also a knee osteoarthritis and at-risk cohort with a relatively low mean depressive-symptom burden, whereas the comparison cohorts are unselected ageing populations. Both differences plausibly contribute to the smaller estimates observed here.

### Limitations

Several limitations should be noted. First, the OAI assesses pain and depression only at intervals of ≥ 12 months (intermediate visits omit the CES-D). Experience-sampling research in knee OA shows that, at the scale of hours to days, within-person pain–affect coupling is present and is moderated by depression [21]; the findings should therefore be interpreted as applying to yearly intervals and do not exclude shorter-term associations. Pain–mood dynamics may operate over days or weeks rather than years, and such shorter-term dynamics are best addressed by intensive longitudinal designs (e.g., diary or ecological momentary assessment). Second, the RI-CLPM is not universally preferable; its suitability depends on the research question and model assumptions. It answers a specifically within-person question and can itself be affected by regression-to-the-mean artefacts. Like the CLPM, the RI-CLPM relies on modelling assumptions and should be interpreted alongside alternative specifications [22]; our interpretation is limited to the distinction between stable between-person variation and year-to-year within-person associations and does not assert that earlier CLPM-based findings are invalid. Third, the equality constraints imposed on the lagged paths were supported on the standardised scale used here but not, for the autoregressive paths, on the pooled scale. The constraint on the cross-lagged paths held on both scales, and the cross-lagged estimates were unchanged when it was relaxed, so this affects the description of stability over time rather than the substantive conclusion.

Several further caveats apply. Cross-lagged effects are not treatment effects, so the absence of a naturalistic within-person prediction does not imply that treating pain or depression is ineffective. The CES-D captures symptom burden rather than a clinical diagnosis, and somatic CES-D items may overlap with pain. Because the worse knee was defined visit by visit, the joint contributing the pain score may change between waves, so the autoregressive paths track overall knee-pain burden rather than a single fixed joint; an index-knee specification would address this and is a worthwhile extension. Finally, attrition across waves is mitigated but not eliminated by FIML and the sensitivity analyses. Generalisability may also be limited, because the OAI is a selected research cohort of adults with, or at elevated risk of, knee OA and may not represent all patients seen in routine clinical care. Throughout, the findings should be read as limited evidence for reciprocal within-person associations at annual intervals rather than as evidence that such associations are absent.

### Clinical implications

If the pain–depression association in knee OA is predominantly between-person, the comorbidity is better understood as a persistent co-occurrence of higher pain and depressive symptom burden than as a year-to-year escalating process. The findings support assessment of depressive symptoms in patients with knee OA, but they do not show that treating one condition will improve the other; this would require confirmation in interventional and finer-grained longitudinal designs. A cross-lagged coefficient describes naturalistic covariation and is not an estimate of a treatment effect; a small cross-lagged path is compatible with a substantial response to intervention. Trials addressing this question would benefit from pre-specifying cross-domain endpoints and from within-person designs that separate treatment-induced change from stable between-person differences. For the coupling the present design cannot resolve, diary or ecological momentary assessment over days to weeks is the appropriate approach [21].

## Conclusions

In the OAI, the association between knee pain and depressive symptoms was primarily explained by stable between-person differences rather than year-to-year within-person effects. The cross-lagged estimates were small, non-significant and robust across a second time window, covariate adjustment and sensitivity analyses. The evidence for reciprocal within-person effects at annual intervals was therefore limited, which is not the same as evidence that such effects are absent. These findings do not exclude shorter-term pain–mood dynamics or treatment effects, but they do not support a strong year-to-year reciprocal feedback loop, and continued assessment of co-occurring depressive symptoms in knee OA remains warranted.

## Supplementary Information

Additional file 1. Title of data: Supplementary tables S1 to S8. Description of data: Table S1, sample formation and wave-specific data availability; Table S2, complete parameter estimates of the primary RI-CLPM; Table S3, wave-specific correlations between WOMAC pain and CES-D total and subscale scores; Table S4, RI-CLPM estimates using the cognitive-affective and somatic CES-D subscales; Table S5, baseline-pain moderation under alternative groupings; Table S6, nested model comparison series for the equality constraints; Table S7, sensitivity of the primary model to the standardisation procedure; Table S8, distributional characteristics at each wave.

## Data Availability

The data analysed in this study were obtained from the Osteoarthritis Initiative (OAI), a publicly accessible research resource (https://nda.nih.gov/oai). Access is available to qualified investigators upon registration and acceptance of the OAI data-use terms. Analysis code is available from the corresponding author on reasonable request.

## Abbreviations

CES-D: Center for Epidemiologic Studies Depression Scale
CFI: Comparative fit index
CLPM: Cross-lagged panel model
FIML: Full-information maximum likelihood
KL: Kellgren-Lawrence
OA: Osteoarthritis
OAI: Osteoarthritis Initiative
RI-CLPM: Random-intercept cross-lagged panel model
RMSEA: Root mean square error of approximation
SRMR: Standardised root mean square residual
WOMAC: Western Ontario and McMaster Universities Osteoarthritis Index
