# Supplementary material for: Re-examining the pain–depression vicious cycle in knee osteoarthritis: a random-intercept cross-lagged panel analysis
Source: BMC Musculoskelet Disord. 2026 Sep 5;27:814. doi: 10.1186/s12891-026-10439-2 (PMC13628889; doi:10.1186/s12891-026-10439-2)
Supplement: Supplementary file 1 [file 12891_2026_10439_MOESM1_ESM.docx]

**Additional file 1: Supplementary tables**

**Re-examining the pain–depression vicious cycle in knee osteoarthritis: a random-intercept cross-lagged panel analysis**

Yannick Bruns, Hendrik Fahlbusch

**Table S1.** Sample formation and wave-specific data availability.

| **Step** | **n** |
| --- | --- |
| Enrolled in the OAI | 4796 |
| No pain or CES-D observation between months 12 and 48 (excluded) | 158 |
| Analysed under full-information maximum likelihood (≥ 1 observation) | 4638 |
| Complete data on all eight panel variables | 3525 |

| **Wave** | **WOMAC pain, n** | **CES-D, n** | **Both, n** | **CES-D missing, n** |
| --- | --- | --- | --- | --- |
| 12 months | 4475 | 4303 | 4300 | 335 |
| 24 months | 4308 | 4131 | 4127 | 507 |
| 36 months | 4256 | 4027 | 4020 | 611 |
| 48 months | 4235 | 4024 | 4013 | 614 |

*Wave-specific counts refer to the analytic sample (n = 4638). Of these, 3925 participants contributed data at all four waves, 337 at three, 212 at two and 164 at a single wave. Missing observations arise from missed visits, incomplete questionnaire return and study attrition; the OAI public-use data do not allow these sources to be distinguished reliably. CES-D, Center for Epidemiologic Studies Depression Scale; WOMAC, Western Ontario and McMaster Universities Osteoarthritis Index.*

**Table S2.** Complete parameter estimates of the primary random-intercept cross-lagged panel model (primary window, months 12 to 48).

| **Parameter** | **Estimate** | **SE** | **z** | **p** | **95% CI** | **Standardised** |
| --- | --- | --- | --- | --- | --- | --- |
| Variance, random intercept pain | 0.654 | 0.017 | 38.34 | < 0.001 | 0.621 to 0.688 | – |
| Variance, random intercept depression | 0.674 | 0.017 | 38.90 | < 0.001 | 0.640 to 0.708 | – |
| Covariance, random intercepts | 0.264 | 0.013 | 20.49 | < 0.001 | 0.239 to 0.289 | 0.39 |
| Variance, within-person pain, wave 1 | 0.368 | 0.011 | 32.96 | < 0.001 | 0.346 to 0.390 | – |
| Variance, within-person depression, wave 1 | 0.366 | 0.011 | 33.36 | < 0.001 | 0.344 to 0.387 | – |
| Covariance, within-person components, wave 1 | 0.018 | 0.008 | 2.20 | 0.028 | 0.002 to 0.033 | 0.048 |
| Autoregression, pain (equal across waves) | 0.161 | 0.017 | 9.28 | < 0.001 | 0.127 to 0.194 | 0.161 |
| Autoregression, depression (equal across waves) | 0.098 | 0.017 | 5.71 | < 0.001 | 0.064 to 0.131 | 0.097 |
| Cross-lagged, pain to depression (equal) | 0.018 | 0.014 | 1.26 | 0.206 | −0.010 to 0.046 | 0.019 |
| Cross-lagged, depression to pain (equal) | 0.009 | 0.014 | 0.62 | 0.538 | −0.019 to 0.037 | 0.009 |
| Residual variance, pain, wave 2 | 0.378 | 0.012 | 32.34 | < 0.001 | 0.355 to 0.401 | – |
| Residual variance, depression, wave 2 | 0.331 | 0.011 | 30.04 | < 0.001 | 0.310 to 0.353 | – |
| Residual covariance, wave 2 | 0.025 | 0.008 | 3.02 | 0.003 | 0.009 to 0.041 | 0.069 |
| Residual variance, pain, wave 3 | 0.350 | 0.011 | 31.71 | < 0.001 | 0.329 to 0.372 | – |
| Residual variance, depression, wave 3 | 0.347 | 0.011 | 30.97 | < 0.001 | 0.325 to 0.369 | – |
| Residual covariance, wave 3 | 0.015 | 0.008 | 1.90 | 0.058 | −0.001 to 0.031 | 0.043 |
| Residual variance, pain, wave 4 | 0.356 | 0.010 | 36.11 | < 0.001 | 0.337 to 0.376 | – |
| Residual variance, depression, wave 4 | 0.369 | 0.011 | 35.12 | < 0.001 | 0.348 to 0.389 | – |
| Residual covariance, wave 4 | 0.026 | 0.007 | 3.50 | < 0.001 | 0.011 to 0.040 | 0.070 |

*Full-information maximum likelihood, n = 4638. Observed variables were z-standardised within wave, so unstandardised estimates are in within-wave standard deviation units. Eight freely estimated intercepts are omitted. The model estimated 27 free parameters, leaving 17 degrees of freedom. Standardised values follow the usual convention of dividing the parameter by the model-implied standard deviations of the variables involved; for equality-constrained paths the mean across transitions is shown. The standardised residual covariances (0.043 to 0.070) correspond to the within-wave residual correlations quoted in the main text. CI, confidence interval; SE, standard error.*

**Table S3.** Concurrent and cross-wave correlations between WOMAC pain and depressive symptoms.

| **Wave** | **n** | **r, CES-D total** | **ρ, CES-D total** | **r, cognitive–affective** | **r, somatic** |
| --- | --- | --- | --- | --- | --- |
| 12 months | 4300 | 0.267 | 0.247 | 0.201 (n = 4278) | 0.311 (n = 4297) |
| 24 months | 4127 | 0.262 | 0.234 | 0.205 (n = 4107) | 0.305 (n = 4126) |
| 36 months | 4020 | 0.262 | 0.251 | 0.212 (n = 3996) | 0.285 (n = 4020) |
| 48 months | 4013 | 0.283 | 0.244 | 0.236 (n = 3997) | 0.304 (n = 4012) |

**Full correlation matrix across all waves (lower triangle)**

|  | **P12** | **D12** | **P24** | **D24** | **P36** | **D36** | **P48** | **D48** |
| --- | --- | --- | --- | --- | --- | --- | --- | --- |
| P12 | – |  |  |  |  |  |  |  |
| D12 | 0.267 | – |  |  |  |  |  |  |
| P24 | 0.675 | 0.255 | – |  |  |  |  |  |
| D24 | 0.238 | 0.686 | 0.262 | – |  |  |  |  |
| P36 | 0.643 | 0.254 | 0.679 | 0.239 | – |  |  |  |
| D36 | 0.231 | 0.640 | 0.237 | 0.678 | 0.262 | – |  |  |
| P48 | 0.619 | 0.241 | 0.629 | 0.232 | 0.698 | 0.247 | – |  |
| D48 | 0.238 | 0.610 | 0.248 | 0.645 | 0.270 | 0.669 | 0.283 | – |

*Pearson (r) and Spearman (ρ) coefficients, computed on complete pairs at each wave; all p < 0.001. The n for the total score equals the number of participants with both measures at that wave (Table S1). Subscale correlations rest on slightly fewer observations, because a subscale score requires at least 80 per cent of its own items to have been answered. The cognitive–affective subscale comprises the thirteen non-somatic CES-D items; the somatic subscale comprises items 1, 2, 5, 7, 11, 13 and 20. In the full matrix, P denotes WOMAC pain and D the CES-D total score, followed by the month of assessment; pairwise complete observations, minimum n = 3790. Within-domain correlations across waves range from 0.619 to 0.698 for pain and from 0.610 to 0.686 for depressive symptoms, indicating substantial temporal stability in both domains. Cross-domain correlations range from 0.231 to 0.287 and show no systematic decline as the interval between waves lengthens, which is the pattern expected if the association reflects stable between-person differences rather than a time-dependent process.*

**Table S4.** Random-intercept cross-lagged panel model using alternative definitions of depressive symptoms.

| **Definition of depressive symptoms** | **Pain → depression** | **Depression → pain** | **Trait r** | **χ² / df** | **CFI** | **RMSEA** | **SRMR** |
| --- | --- | --- | --- | --- | --- | --- | --- |
| CES-D total score (primary) | 0.019 (p = 0.21) | 0.009 (p = 0.54) | 0.39 | 18.4 / 17 | 1.000 | 0.004 | 0.006 |
| Cognitive–affective items only | 0.014 (p = 0.35) | −0.001 (p = 0.95) | 0.34 | 19.3 / 17 | 1.000 | 0.005 | 0.007 |
| Somatic items only (contrast) | 0.028 (p = 0.053) | 0.025 (p = 0.071) | 0.44 | 21.7 / 17 | 1.000 | 0.008 | 0.008 |

*Standardised cross-lagged coefficients, primary window (months 12 to 48), full-information maximum likelihood. Unstandardised estimates for the cognitive–affective model: pain to depression 0.014 (SE 0.015); depression to pain −0.001 (SE 0.013). For the somatic model: 0.029 (SE 0.015) and 0.025 (SE 0.014). Trait r, correlation between the two random intercepts.*

**Table S5.** Moderation of the within-person cross-lagged paths by baseline pain: groupings alternative to the median split.

| **Grouping of baseline WOMAC pain** | **LRT χ²** | **df** | **p** | **Group-specific estimates (pain → depression / depression → pain)** |
| --- | --- | --- | --- | --- |
| > 0 versus 0 (any pain) | 1.39 | 2 | 0.499 | n = 1279: +0.019 / −0.014; n = 3359: +0.018 / +0.013 |
| ≥ 5 versus < 5 (upper third) | 0.24 | 2 | 0.886 | n = 3220: +0.019 / +0.009; n = 1418: +0.025 / +0.019 |
| 0 / 1 to 3 / ≥ 4 (three groups) | 2.60 | 4 | 0.627 | n = 1279: +0.019 / −0.014; n = 1597: +0.038 / +0.033; n = 1762: +0.020 / +0.013 |

*Likelihood-ratio tests comparing multigroup models with freely estimated versus equality-constrained cross-lagged paths; 2 degrees of freedom for two-group and 4 for three-group comparisons. Group-specific coefficients are standardised. Baseline pain refers to the worse-knee WOMAC pain score at the OAI baseline visit (median 2, interquartile range 0 to 5). The primary median split is reported in Table 4 of the main text and is not repeated here.*

**Table S6.** Nested model comparison series evaluating the equality constraints.

**Within-wave standardisation (primary specification)**

| **Model** | **χ²** | **df** | **CFI** | **TLI** | **RMSEA** | **SRMR** | **AIC** | **BIC** | **LRT vs primary** |
| --- | --- | --- | --- | --- | --- | --- | --- | --- | --- |
| Primary: autoregressive and cross-lagged paths constrained, within-wave residual covariances free | 18.41 | 17 | 1.000 | 1.000 | 0.004 | 0.006 | 77801 | 77975 | – |
| Autoregressive paths freed | 13.05 | 13 | 1.000 | 1.000 | 0.001 | 0.006 | 77804 | 78004 | p = 0.253 |
| Cross-lagged paths freed | 15.95 | 13 | 1.000 | 1.000 | 0.007 | 0.005 | 77807 | 78006 | p = 0.652 |
| Within-wave residual covariances additionally constrained | 19.63 | 19 | 1.000 | 1.000 | 0.003 | 0.006 | 77798 | 77960 | p = 0.543 |
| All parameters freed | 10.12 | 9 | 1.000 | 1.000 | 0.005 | 0.005 | 77809 | 78034 | p = 0.406 |

**Pooled standardisation (equivalent to the raw scale)**

| **Model** | **χ²** | **df** | **CFI** | **TLI** | **RMSEA** | **SRMR** | **AIC** | **BIC** | **LRT vs primary** |
| --- | --- | --- | --- | --- | --- | --- | --- | --- | --- |
| Primary specification | 32.74 | 17 | 0.999 | 0.999 | 0.014 | 0.008 | 77772 | 77946 | – |
| Autoregressive paths freed | 16.61 | 13 | 1.000 | 1.000 | 0.008 | 0.008 | 77764 | 77964 | p = 0.003 |
| Cross-lagged paths freed | 29.63 | 13 | 0.999 | 0.998 | 0.017 | 0.007 | 77777 | 77977 | p = 0.539 |
| Within-wave residual covariances additionally constrained | 34.04 | 19 | 0.999 | 0.999 | 0.013 | 0.009 | 77769 | 77931 | p = 0.522 |
| All parameters freed | 10.59 | 9 | 1.000 | 1.000 | 0.006 | 0.005 | 77766 | 77992 | p = 0.005 |

*The primary specification constrains the autoregressive and cross-lagged paths to equality across waves while leaving the within-wave residual covariances free; each subsequent row relaxes or adds one constraint relative to it, and the likelihood-ratio test compares that model with the primary specification. Under within-wave standardisation no constraint is rejected. Under pooled standardisation the constraint on the autoregressive paths is rejected; those paths increase across waves (pain: 0.118, 0.125, 0.195). The constraint on the cross-lagged paths is supported under both standardisations. Cross-lagged estimates were unaffected: with the autoregressive paths freed, the standardised cross-lagged coefficients were 0.020 and 0.011 under within-wave standardisation and 0.022 and 0.010 under pooled standardisation; with all six cross-lagged paths freely estimated they ranged from −0.030 to +0.045, none significant. AIC, Akaike information criterion; BIC, Bayesian information criterion; LRT, likelihood-ratio test.*

**Table S7.** Sensitivity of the primary model to the standardisation procedure.

| **Standardisation** | **χ² / df** | **CFI** | **RMSEA** | **SRMR** | **Pain → depression** | **Depression → pain** | **Trait r** |
| --- | --- | --- | --- | --- | --- | --- | --- |
| Within-wave (primary) | 18.41 / 17 | 1.000 | 0.004 | 0.006 | 0.018 (p = 0.21) | 0.009 (p = 0.54) | 0.39 |
| Pooled across waves | 32.74 / 17 | 0.999 | 0.014 | 0.008 | 0.018 (p = 0.20) | 0.007 (p = 0.63) | 0.39 |

*Standardised cross-lagged coefficients. Pooled standardisation applies a single mean and standard deviation per construct across all waves and is therefore a uniform linear transformation, equivalent to analysing the raw WOMAC and CES-D scales. Model fit is more favourable under within-wave standardisation because wave-specific differences in variance are absorbed by the transformation rather than modelled; the substantive estimates and the between-person trait correlation are unchanged.*

**Table S8.** Distributional characteristics of WOMAC pain and CES-D at each analysed wave.

| **Wave** | **Measure** | **n** | **Mean (SD)** | **Median (IQR)** | **Skewness** | **Kurtosis** |
| --- | --- | --- | --- | --- | --- | --- |
| 12 months | WOMAC pain | 4475 | 3.01 (3.58) | 2 (0–5) | 1.42 | 1.67 |
| 12 months | CES-D | 4303 | 6.65 (7.15) | 4 (1–9) | 1.93 | 5.10 |
| 24 months | WOMAC pain | 4308 | 2.93 (3.50) | 2 (0–5) | 1.46 | 1.95 |
| 24 months | CES-D | 4131 | 6.23 (6.96) | 4 (1–9) | 2.03 | 5.64 |
| 36 months | WOMAC pain | 4256 | 2.92 (3.58) | 2 (0–5) | 1.50 | 1.96 |
| 36 months | CES-D | 4027 | 6.61 (7.22) | 4 (1–9) | 1.93 | 4.73 |
| 48 months | WOMAC pain | 4235 | 2.91 (3.55) | 2 (0–5) | 1.46 | 1.82 |
| 48 months | CES-D | 4024 | 6.97 (7.49) | 4 (2–10) | 1.77 | 3.69 |

*Both measures are right-skewed at every wave. Table 1 of the main text reports the corresponding summaries for the first analysed wave. WOMAC pain refers to the worse knee.*
